# Coronaviruses in Children, Greece

**DOI:** 10.3201/eid1306.061353

**Published:** 2007-06

**Authors:** Anna Papa, Evangelia Papadimitriou, Luciano Kleber de Souza Luna, Motassim Al Masri, Efimia Souliou, Maria Eboriadou, Antonis Antoniadis, Christian Drosten

**Affiliations:** * Aristotle University of Thessaloniki, Thessaloniki, Greece; † Berhard Nocht Institute for Tropical Medicine, Hamburg, Germany; ‡ University General AHEPA Hospital, Thessaloniki, Greece

**Keywords:** Coronavirus, HCoV-229E, HCoV-OC43, HCoV-NL63, children, respiratory infection, Greece, letter

**To the Editor:** Two recently detected human coronaviruses (HCoVs), NL63 and HKU1, increased the number of coronaviruses known to infect humans to 5 (*1*–*3*). HCoV-229E and HCoV-NL63 belong to antigenic group 1, HCoV-OC43 and HCoV-HKU1 belong to antigenic group 2, and severe acute respiratory syndrome (SARS)–associated coronavirus (SARS-CoV) is most closely related to group 2 coronaviruses. In 2005, an optimized pancoronavirus reverse transcription–PCR assay was used to explore the incidence of HCoV-NL63 infection in children in Belgium who had a diagnosis of respiratory tract infection (*4*). We report the results of an epidemiologic study that used a universal coronavirus RT-PCR assay to detect coronaviruses among children in Greece with acute respiratory tract infections.

We tested throat swab specimens obtained from children hospitalized in Greece during June 2003 through May 2004 (200 children 2 months to 14 years of age, mean 4.09 years) and during December 2005 through March 2006 (44 children 1.6–8.5 years of age, mean 5.05). Specimens were obtained the first day of each child’s hospitalization, and all specimens were included in the study, regardless whether other respiratory microorganisms were detected.

The 25-μL reaction contained 200 µM dNTPs, 0.2 µM primer PC2S2 (equimolar mixture of 5′-ttatgggttgggattatc-3′ and 5′-tgatgggatgggactatc-3′), 0.8 µM primer PC2As1 (5′-tcatcagaaagaatcatca-3′), 1 μL of enzyme mix from the QIAGEN One-Step RT-PCR Kit (QIAGEN GmbH, Hilden, Germany), and 5 μL of RNA. The initial 30-min reverse transcription step at 48°C was followed by 10 cycles of 20 sec at 94°C, 30 sec at 62°C with a decrease of 1°C per cycle, 40 sec at 72°C; 40 cycles of 20 sec at 94°C, 30 sec at 52°C, 40 sec at 72°C; and a final extension step at 72°C for 10 min. To determine the sensitivity after optimization, we tested quantified RNA in vitro transcripts that included the natural primer binding sites of the respective coronavirus genomes. Sensitivities for SARS-CoV, HCoV-OC43, HCoV-229E, and HCoV-NL63 were 61.0, 800.0, 8.2, and 82.3 nominal RNA copies per assay, respectively. A separate test was not done for HCoV-HKU1 because it had the same primer binding sites as HCoV-OC43. A phylogenetic tree based on a 400-bp genome fragment of the polymerase gene was constructed (Appendix Figure).

Of 200 samples collected in 2003–2004, 5 (2.5%) were positive for coronaviruses (2 each for HCoV-NL63 and HCoV-229E and 1 for HCoV-OC43), and of 44 samples collected in 2005–2006, 2 (4.5%) were positive for coronaviruses (1 for HCoV-229E and 1 for HCoV-OC43) (GenBank accession nos. EF103180–EF103184, EF394298, and EF394299). CoV-HKU1 was not detected.

The amplified genome region is one of the most conserved regions of the coronavirus genome. However, sequences for HCoV-NL63 strains isolated in Greece are genetically closer to the sequence for a strain (AY567487) isolated in Amsterdam in 2003 (*1*) than to a strain (AY518894) from a specimen collected in Rotterdam in 1988 (*2*) (0.6% vs. 1.1% nucleotide divergence). Sequences for HCoV-229E and HCoV-OC43 strains isolated in Greece differ from sequences for strains isolated elsewhere by 0.5%–1.7%.

The HCoV-NL63–positive specimens in our study were obtained from a 9- and a 14-month-old child during winter 2003–2004; no cases were identified during 2005–2006. Specimens positive for HCoV-229E and HCoV-OC43 were detected during both study periods (Table). HCoV-OC43 affected children with a mean age of 3.1 years (median, 1.4 years), and HCoV-229E affected children with a mean (and median) age of 5.5 years. However, no general conclusions can be drawn from these data because number of cases is too few.

**Table:** Epidemiologic and laboratory data for patients with coronavirus infection, Greece*

Specimen no., HCoV strain	Age, sex	Sample date	Symptoms	WBC (cells/ mm^3^)	Granulocytes, %	ESR (mm/h)	Days in hospital	Coinfection
10/03, 229E	3 y, F	Jun 3, 2003	Fever (39°C), cough, pharyngitis	10,400	87	40	3	RSV
16/03, 229E	8 y, M	Jun 14, 2003	Fever (41°C), headache, rhinitis, sinusitis	18,900	86.4	30	4	ND
109/03, NL63	14 mo, F	Nov 30, 2003	Fever (39°C), cough, severe pneumonia	18,700	44.0	85	12	ND
173/04, NL63	10 mo, M	Feb 10, 2004	Fever (38.5°C), cough, rhinitis, tachypnea, bronchiolitis	7,100	57.9	55	3	ND
185/04, OC43	17 mo, F	Feb 25, 2004	Pharyngitis, rhinitis, respiratory distress, bronchiolitis	10,100	63.2	30	2	ND
12A/06, OC43	6 mo, F	Jan 11, 2006	Fever (38.8°C), cough, tachypnea, bronchiolitis	19,950	80.3	35	6	RSV
14A/06, 229E	7.5 y, M	Feb 13, 2006	Fever (40.5°C), cough, rhinitis	20,600	83.1	98	4	*Mycoplasma pneumoniae*

*HCoV, human coronavirus; WBC, white blood cell count; ESR, erythrocyte sedimentation rate; RSV, respiratory syncytial virus; ND, not detected.

None of the patients in Greece had an underlying disease, and all recovered completely. Patients infected with HCoV-229E had been hospitalized for upper respiratory tract infections, and those with HCoV-OC43 had lower respiratory tract infections; all cases were mild. Both children infected with HCoV-NL63 had symptoms of lower respiratory tract infections: 1 child had severe pneumonia and was hospitalized for 12 days, while the other had a mild course of bronchiolitis.

HCoV-NL63 was first identified in Amsterdam, the Netherlands, by van der Hoek et al. (*1*) from a nasopharyngeal specimen obtained in 2003 from a 7-month-old child with bronchiolitis, conjunctivitis, and fever. One month later, Fouchier et al. (*2*) reported the characterization of the same virus isolated from a specimen collected in 1988. The specimen had been obtained from an 8-month-old child with pneumonia in Rotterdam, the Netherlands. Later, HCoV-NL63 was detected in 2.5% of bronchiolitis patients <2 years of age in Japan (*5*) and in most children hospitalized with bronchiolitis in Australia and Canada (*6*,*7*).

Coinfection with HCoVs and other respiratory viruses is frequently observed and is associated with severe clinical syndromes, especially in infants and young children (*6*,*8*). Coinfection was observed in 3 of the 7 HCoV-positive patients in our study. The 3 patients were infected with HCoV-OC43 or HCoV-229E; coinfection with respiratory syncytial virus was found in 2 patients, and coinfection with *Mycoplasma pneumoniae* was found in 1 patient. It was not possible to determine the role of the HCoVs in these coinfections. In addition, because coronaviruses can be detected even 3 weeks after an acute episode, some cases of coinfection might represent former rather than current HCoV infection (*9*).

In conclusion, we detected 3 types of HCoVs in Greece: 229E, OC43, and NL63. This finding provides initial insight into the epidemiologic features of coronaviruses in Greece. Further studies are needed to find the exact clinical effect of these HCoVs in humans and to elucidate the epidemiology of coronaviruses worldwide.

## Supplementary Material

Appendix Figure Phylogenetic tree based on a 400-bp genome fragment of the polymerase gene of coronaviruses, including sequences from the present study (in boldface). Numbers at nodes represent the percentage of 100 bootstrap replicates that contained the cluster distal to the node. Bootstrap values >60% are indicated. HCoV, human coronavirus; SARS, severe acute respiratory syndrome.
