# Supplementary figures and images for: Coronaviruses in Children, Greece
Source: Emerg Infect Dis. 2007 Jun;13(6):947–9. doi: 10.3201/eid1306.061353 (PMC2792836; doi:10.3201/eid1306.061353)

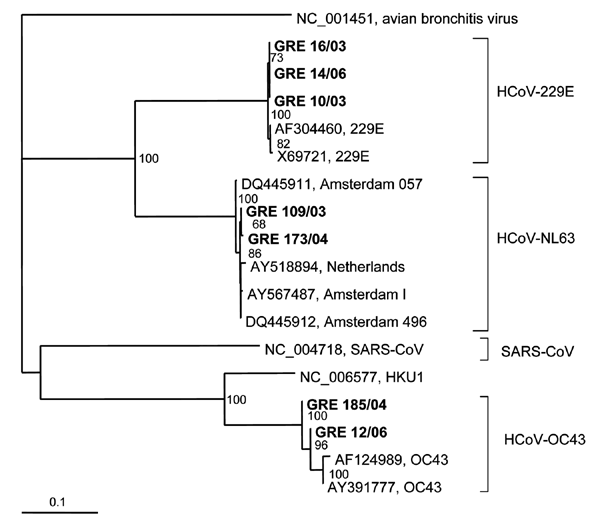

Supplement: Appendix Figure — Phylogenetic tree based on a 400-bp genome fragment of the polymerase gene of coronaviruses, including sequences from the present study (in boldface). Numbers at nodes represent the percentage of 100 bootstrap replicates that contained the cluster distal to the node. Bootstrap values >60% are indicated. HCoV, human coronavirus; SARS, severe acute respiratory syndrome. [file 06-1353_appF-s1.gif]
